# Hat1 acetylates histone H4 and modulates the transcriptional program in *Drosophila* embryogenesis

**DOI:** 10.1038/s41598-019-54497-0

**Published:** 2019-11-29

**Authors:** Júlia Varga, Szabina Korbai, Alexandra Neller, Nóra Zsindely, László Bodai

**Affiliations:** 1 Department of Biochemistry and Molecular Biology, Faculty of Science and Informatics, University of Szeged, 6726 Szeged, Közép fasor 52, Hungary; 2 Doctoral School in Biology, Faculty of Science and Informatics, University of Szeged, 6726 Szeged, Hungary

**Keywords:** Histone post-translational modifications, Gene expression, Transcriptomics

## Abstract

Post-translational modifications of histone proteins play a pivotal role in DNA packaging and regulation of genome functions. Histone acetyltransferase 1 (Hat1) proteins are conserved enzymes that modify histones by acetylating lysine residues. Hat1 is implicated in chromatin assembly and DNA repair but its role in cell functions is not clearly elucidated. We report the generation and characterization of a *Hat1* loss-of-function mutant in *Drosophila*. *Hat1* mutants are viable and fertile with a mild sub-lethal phenotype showing that *Hat1* is not essential in fruit flies. Lack of Hat1 results in the near complete loss of histone H4 lysine (K) 5 and K12 acetylation in embryos, indicating that Hat1 is the main acetyltransferase specific for these marks in this developmental stage. We found that Hat1 function and the presence of these acetyl marks are not required for the nuclear transport of histone H4 as histone variant His4r retained its nuclear localization both in *Hat1* mutants and in His4r-K5R-K12R double point mutants. RNA-seq analysis of embryos indicate that in *Hat1* mutants over 2000 genes are dysregulated and the observed transcriptional changes imply a delay in the developmental program of gene expression.

## Introduction

The hereditary material of eukaryotes can be found in the nucleus in the form of chromatin: a complex of DNA, RNA and protein molecules. The basic building units of chromatin are the nucleosomes, protein octamers containing two copies of each of the histone proteins H2A, H2B, H3 and H4^1,2^. Histones can undergo several post-translational modifications (PTMs), including acetylation, methylation or phosphorylation that might influence transcription and other chromatin templated processes^3^. Histone acetylation that occurs on lysine (K) residues is positively associated with transcription and might exert its effect by charge neutralization and by providing binding surfaces to regulatory proteins^4^. Although histone acetylation mostly occurs in nucleosomal context, nascent pre-nucleosomal histones can also be acetylated^4–6^. These acetyl marks are then shortly removed after replication-coupled chromatin assembly^7,8^. Newly synthesized H4 histones are diacetylated on residues K5/K12 in several diverse organisms including *Tetrahymena*, *Drosophila*, chicken, mouse and humans^6,9,10^. As H4 acetyl-K5/K12 pre-deposition marks are rapidly removed after chromatin assembly^8,10^ it seems feasible that the K5/K12 diacetyl mark might play important roles in nuclear transport of histones and/or chromatin assembly/maturation. However, experimental results only partially supported these hypotheses^11–13^.

The well conserved H4 K5/K12 diacetylation mark is generated by cytoplasmic histone acetyltransferase 1 (HAT1) enzymes. HAT1 was first described in *S. cerevisiae* as an acetyltransferase showing activity towards the H4K12 residues^14^. Loss-of function mutation of *Hat1*, however, did not cause growth defects or any other apparent phenotypes^14^. HAT1 forms a catalytically active holoenzyme with a second protein, HAT2 that shows homology to mammalian Rbap46 and Rbap48 proteins^15^. HAT2 facilitates the binding of HAT1 to the histone H4 tail and dramatically increases its acetyltransferase activity^15^. Similarly, in human cells HAT1 can be found in a heterodimeric holoenzyme with p46/RBBP7 (Retinoblastoma-binding protein 7), a protein highly homologous to yeast HAT2^16^. In contrast to yeast cells, Hat1 is indispensable in mice since it is essential for proper development. *Hat1* null pups that are born show lung and cranial developmental defects and die shortly after birth^10^. In cultured murine cells Hat1 is required for the acetylation of nascent histones, illustrated by a decrease in the acetylation levels of H4K5 and H4K12 in *Hat1* null cells. Surprisingly, similar change was also observed in the case of H3K9, H3K18 and H3K27 that are incorporated during replication. Loss of *Hat1* also leads to decreased proliferation rate, decreased genome stability and sensitivity to a wide range of DNA damaging agents in cultured murine cells^10^.

Previous studies suggested that in *Drosophila melanogaster* Hat1 might have functions in formation of centromeric chromatin. In flies Hat1 is most abundant in the cytoplasm, but it can also be detected in nuclear and chromatin fractions^17^. A small portion of Hat1 is associated with the centromeric histone H3 variant Centromeric Protein-A (CENP-A) both in the cytoplasm and in the nucleus^17^. Furthermore, Hat1 was found to be enriched in CENP-A containing chromatin, and in addition to euchromatic sites it was also localized to centromeres during interphase but not during mitosis^18^.

Here we report generation of *Hat1* loss-of-function mutants in *Drosophila* and the phenotypic and molecular analysis of fruit flies lacking Hat1 activity. We show that although Hat1 is the major H4K5 and H4K12 specific acetyltransferase in embryos and its loss results in altered transcript levels of more than 2000 genes it is not essential for viability, fertility or nuclear localization of histone H4 or His4r. Loss of Hat1 does not affect inspected adult phenotypes such as daily activity, sleep pattern, or heat-stress tolerance but mitigates proteopathic stress induced neurodegeneration in a Huntigton’s disease model.

## Results

### Generation of Hat1 mutants

**Figure 1:**
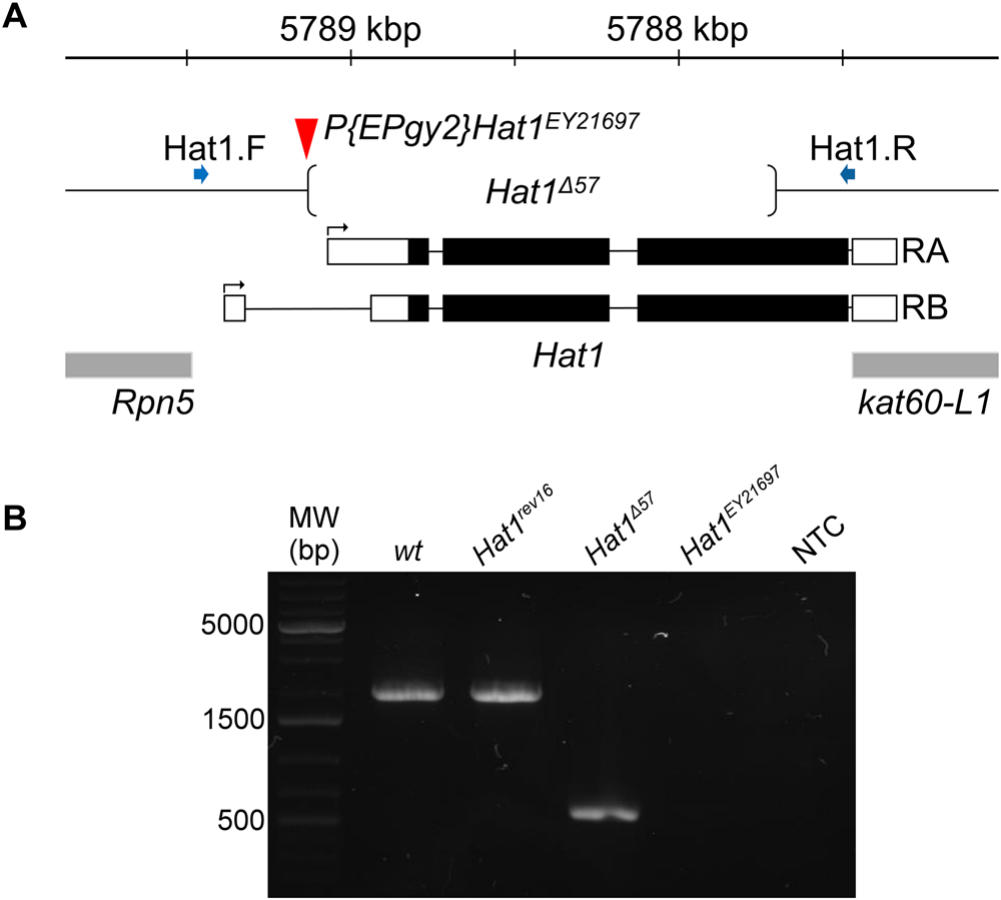
Generation of Hat1 deletion mutant. (**A**) The genomic region of *Hat1* on chromosome 3R is shown under a kilobase scale. On the *Hat1* transcript models black bars represent coding exons, empty bars show untranslated exonic regions, introns are represented by lines, arrows indicate transcriptional start sites. Neighboring genes are shown with grey bars. Red arrowhead marks the position of the P{EPgy2}Hat1^EY21697^ element used for mutagenesis, the breakpoints of the *Hat1*^*Δ57*^ deletion are shown by round brackets. Position of PCR primers are marked by blue arrows. (**B**) Agarose gel electrophoresis of PCR products amplified from wild-type and *Hat1*^*rev16*^ genomic DNA template with Hat1.F and Hat1.R primers show ~2 kbp bands. PCR on *Hat1*^*Δ57*^ template produce a fragment of approximately 600 bp while there is no PCR product from reactions with *P{EPgy2}Hat1*^*EY21697*^ template (having the 10908 bp long transposon inserted) or without template (NTC).

To be able to analyze the functions of Hat1 *in vivo* we generated loss of function *Hat1* mutants. Several transposon insertion alleles of *Hat1* were reported previously but none of these affect the coding region of the gene, therefore cannot be considered null alleles. We chose one of these alleles, P{EPgy2}Hat1^EY21697^ to generate *Hat1* deletion mutants by P element jump-out. P{EPgy2}Hat1^EY21697^ is inserted in the first intron of *Hat1* at genome position 3R:5,789,135 (Fig. 1A). The P{EPgy2} element carries Scer/UAS binding sites in correct orientation to overexpress *Hat1* if a GAL4 driver is present. To remobilize the element we crossed P{EPgy2}Hat1^EY21697^ females to males carrying a third chromosomal Δ2-3 transposase source and selected flies that lost the transposon by screening for loss of the dominant mini-white marker in the second generation. Revertant lines were genotyped by PCR using primers Hat1.F and Hat1.R that straddle the insertion point of P{EPgy2}Hat1^EY21697^ and the coding sequence of *Hat1* and produce a 2007 bp amplicon on wild-type template. Since Hat1 is located in a gene rich genomic region and partially overlaps with a neighboring gene we designed the primers to enable the identification of deletions affecting only *Hat1*: Hat1.F is situated in the intergenic region between Hat1 and its upstream neighbor, *Rpn5*, while Hat1.R is located at the end of the coding sequence of Hat1 juxtaposed to the 3′ UTR of the partially overlapping gene, *kat-60L1*. We tested 226 individual revertants and found 5 which gave a shorter PCR product than the wild-type template. We selected revertant line *Hat1*^*Δ57*^ that gave the shortest PCR amplicon of ~600 bp, therefore having the largest deletion of ~1400 bp, for further analysis (Fig. 1B). We used Sanger sequencing to determine the exact breakpoints of the *Hat1*^*Δ57*^ deletion (Fig. 1A). In *Hat1*^*Δ57*^ a 1409 bp deletion (3 R:5787709-5789127) removes the downstream Hat1-RA promoter, all splice sites and 80% of the coding region (326/405 codons) including the acetyltransferase domain rendering *Hat1*^*Δ57*^ a catalytically null allele. We also selected a precise revertant, *Hat1*^*rev16*^, that did not cause a deletion in the gene (Fig. 1B) to use as control.

### Hat1 is essential for H4K5 and H4K12 acetylation but not required for nuclear localization of H4

**Figure 2:**
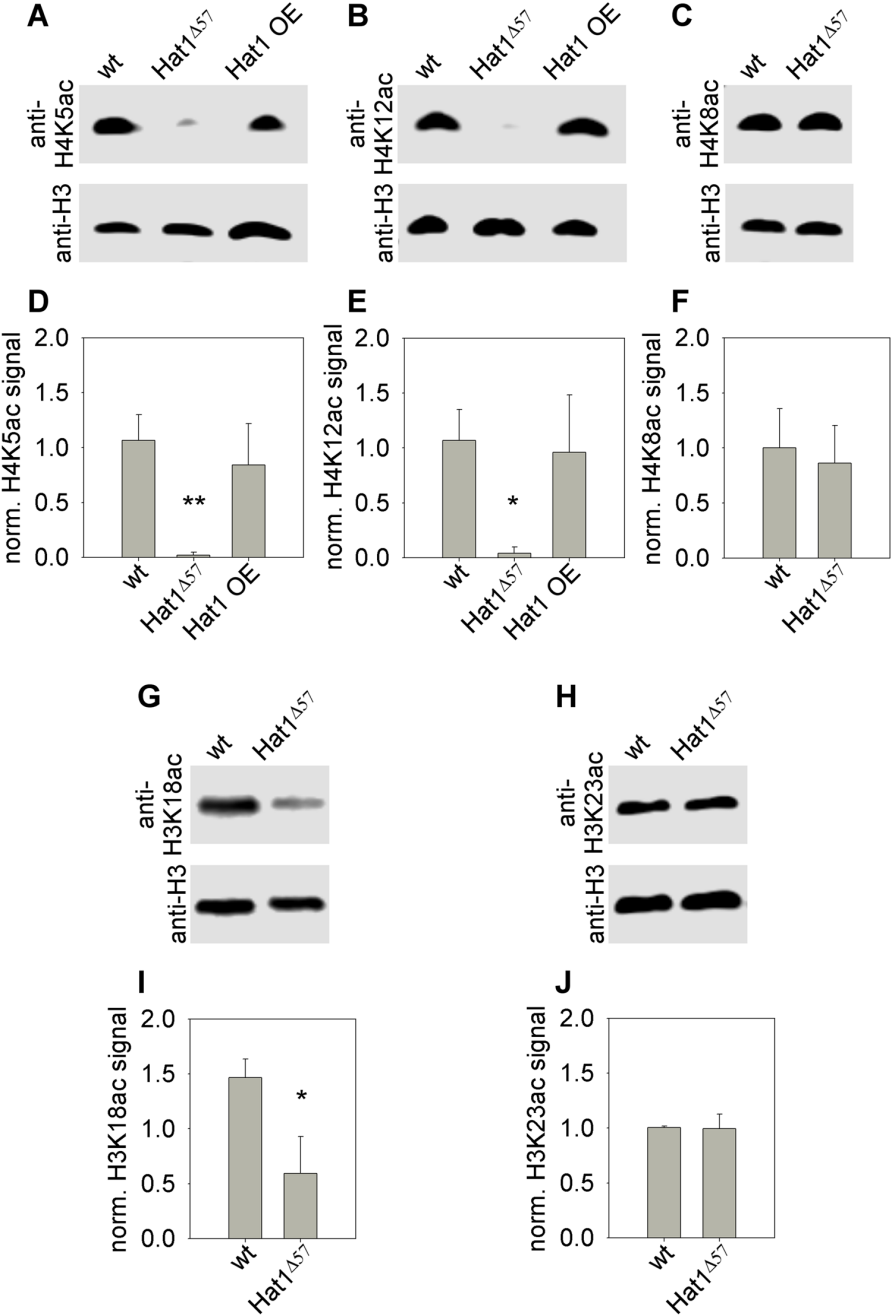
Hat1 acetylates H4K5 and H4K12 residues in embryos. Immunoblot analysis of embryo lysates with antibodies against acetylated histones show that in *Hat1*^*Δ57*^ mutants the level of acetylated H4K5 (**A,D**) and H4K12 (**B,E**) residues is significantly reduced while overexpression of *Hat1* does not affect H4K5ac and H4K12ac levels. Acetylation of H4K8 is not altered in *Hat1*^*Δ57*^ mutants (**C,F**). The level of H3K18 acetylation is lower in *Hat1*^*Δ57*^ embryos than in controls (**G,I**), while there is no change in the abundance of acetyl-H3K23 (**H,J**). Bar graphs show average signal intensities of acetylated histone bands normalized to signal intensities of H3 loading controls, whiskers indicate standard deviation. Asterisks mark significant differences compared to wild-type control, *P < 0.05, **P < 0.01.

Hat1 enzymes were demonstrated to possess H4 specific acetyltransferase activities in several organisms including yeast, mice and humans. Therefore, we aimed to determine whether *Drosophila* Hat1 also has H4 specific acetyltransferase activity. We collected 0–6 hours old *w*^1118^ (wild-type control), *Hat1*^*Δ57*^ and *da-GAL4/Hat1*^*EY21697*^ (*Hat1* overexpressing, Supplementary Fig. S1) embryos for immunoblot analysis. Using acetyl-H4K5 (Fig. 2A,D, Supplementary Fig. S2) and acetyl-H4K12 (Fig. 2B,E, Supplementary Fig. S3) specific antibodies we found that the level of these PTMs diminished significantly in *Hat1*^*Δ57*^ mutants to 1.8% (P = 0.0079, Kruskal-Wallis Test followed by Wilcoxon Rank Sum Test, n = 5) and to 3.95% (P = 0.025, ANOVA with Tukey HSD Test, n = 3), respectively, to that of the wild-type control. These results suggest not only that Hat1 is capable of acetylating H4K5 and H4K12 residues but also that it is the main H4K5 and H4K12 specific acetyltransferase of *Drosophila* in this developmental stage. Surprisingly, however, overexpression of *Hat1* did not result in increased acetylation of either residues (Fig. 2A,B,D,E) suggesting that Hat1 is not a rate limiting component in this catalytic process. As a control experiment, we also analyzed the acetylation level of the H4K8 residue by immunoblotting *Hat1*^*Δ57*^ mutants and found no significant change in the level of this PTM in *Hat1*^*Δ57*^ mutants (Fig. 2C,F, Supplementary Fig. S4). To rule out the possibility that the observed reduction in H4K5 and H4K12 acetylation levels is not due to loss of Hat1 but is caused by background mutations we performed immunoblots on extracts of heterozygotes of *Hat1*^*Δ57*^ and two other *Hat1* imprecise excision mutants and found that acetyl-H4K5 and acetyl-H4K12 levels were severely diminished in these heterozygotes (P < 0.01 in both cases, Kruskal-Wallis Test, n ≥ 3, Supplementary Fig. S5). It was previously shown that in murine cells steady state and replication dependent acetylation levels of specific lysine residues of histone H3 are reduced in the absence of Hat1^10^. Therefore, we also tested the acetylation state of H3K18 (Fig. 2G,I, Supplementary Fig. S6) and H3K23 (Fig. 2H,J, Supplementary Fig. S7) residues in *Hat1*^*Δ57*^ and control embryos by immunoblotting. We found that the level of acetyl-H3K18 was significantly lower in *Hat1*^*Δ57*^ mutants than in wild-type control (40.6% of control, P = 0.028, Welch’s t-test, n = 3) while the level of acetyl-H3K23 did not change.

**Figure 3:**
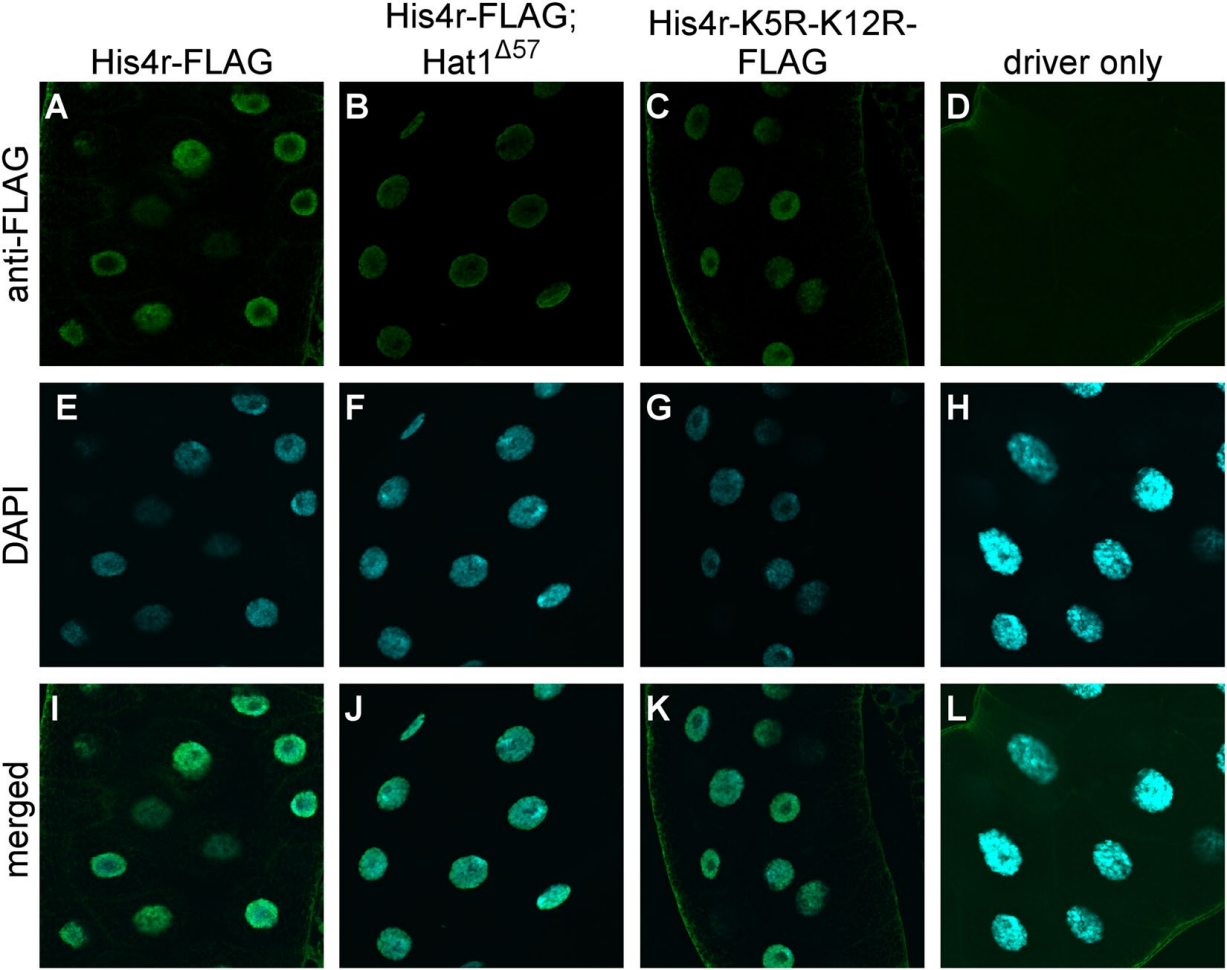
Hat1 dependent H4K5 and H4K12 acetylation is not required for nuclear localization of His4r. His4r protein localizes to the cell nucleus as shown by anti-FLAG immunostaining of polytene larval salivary gland cells expressing transgenic His4r with a C-terminal FLAG tag (**A,I**), nuclei are visualized by DAPI staining (**E**). Nuclear localization of His4r is unaltered in homozygous *Hat1*^*Δ57*^ larvae lacking functional Hat1 (**B,F,J**). Mutated His4r proteins in which Hat1 target lysines were changed to arginines (His4r-K5R-K12R) are also correctly localized to the nucleus (**C,G,K**). In the negative control having the driver element but lacking a His4r-FLAG transgene there is no specific staining with anti-FLAG antibody (**D,H,L**).

As the cytoplasmic acetylation of H4 and His4r histones precedes their nuclear transport we investigated whether H4K5 and H4K12 acetylation by Hat1 is required for nuclear localization of histone H4. For these tests we generated transgenic *Drosophila* strains carrying an inducible His4r transgene with a C-terminal FLAG tag (UAS-His4r) or its mutated derivative in which K5 and K12 lysines were changed to arginine (UAS-His4r-K5R-K12R) mimicking unmodified lysine that is not (and cannot be) acetylated. The amino acid sequence of the protein encoded by the *His4r* gene is identical to the sequence of canonical H4, thus the proteins produced from these transgenes correspond to the histones encoded by both *His4r* and *H4*. To visualize the localization of His4r in the presence or absence of Hat1 we performed immunostaining of *w; hs-GAL4/*+; *Hat1*^*Δ57*^ (staining control, Fig. 3D,H,L), *w; hs-GAL4/*+; *UAS-His4r* (Fig. 3A,E,I), and *w; hs-GAL4/*+; *UAS-His4r Hat1*^*Δ57*^*/Hat1*^*Δ57*^ (Fig. 3B,F,J) larval tissues with anti-FLAG antibody. We found that His4r protein localized to the nucleus both in larvae wild-type for *Hat1* and in homozygous *Hat1*^*Δ57*^ mutants. Having found that Hat1 is not required for the nuclear localization of His4r we investigated whether acetylation of Hat1 target lysines, H4K5 and H4K12, influences the localization of His4r by immunostaining *w; hs-GAL4/*+; *UAS-His4r-K5R-K12R* larvae with anti-FLAG antibody (Fig. 3C,G,K). We found that His4r-K5R-K12R that cannot be acetylated at the mutated positions has a nuclear localization similar to wild-type His4r, suggesting that these PTMs do not have a crucial role in the nuclear transport of His4r and H4 histones.

### *Drosophila Hat1* is not required for viability or fertility

**Figure 4:**
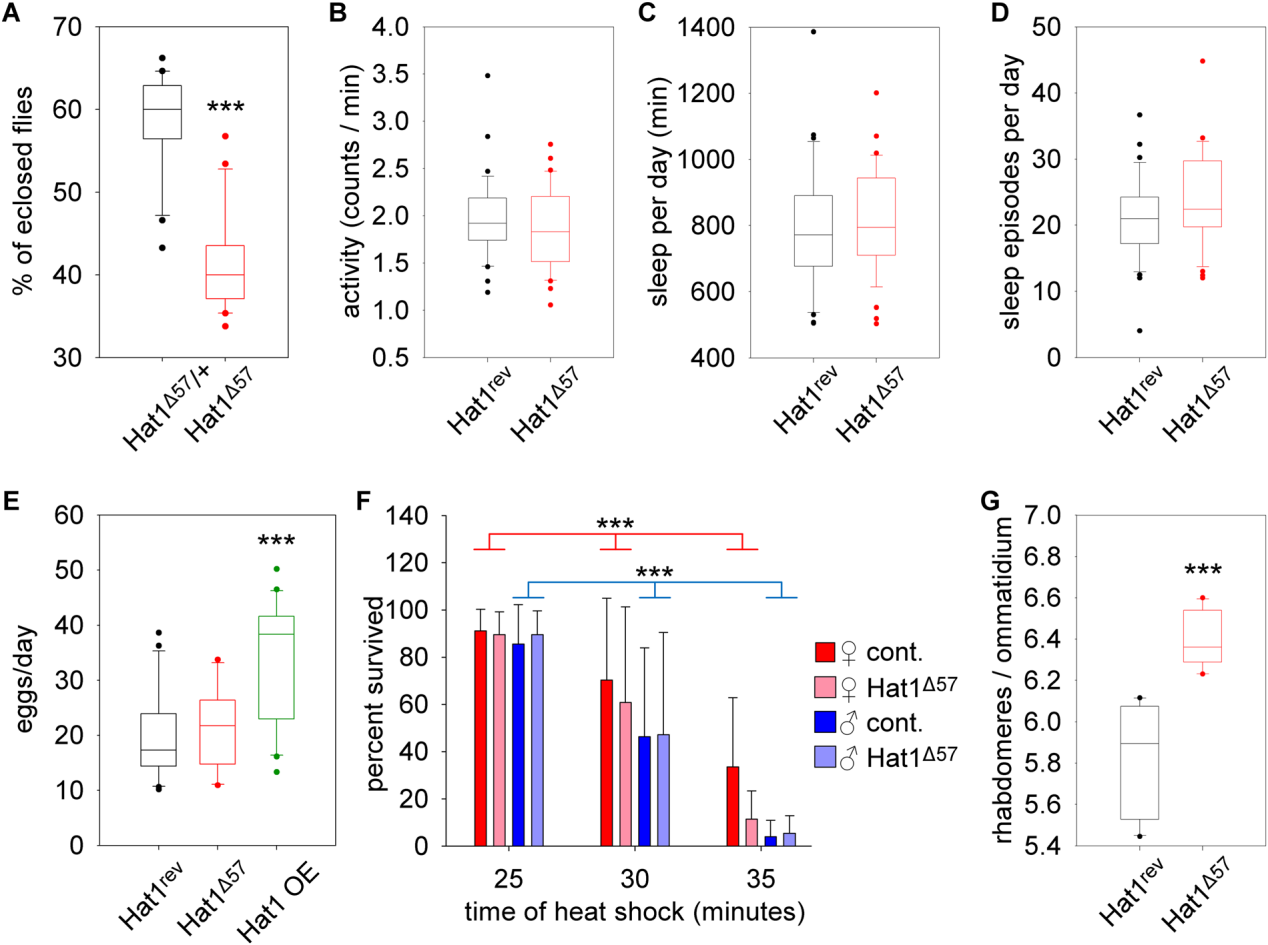
Phenotype analysis of *Hat1* mutants. (**A**) *Hat1*^*Δ57*^ *Drosophila* have reduced viability. The graph shows the percentages per vials of *Hat1*^*Δ57*^*/+* heterozygous and *Hat1*^*Δ57*^ homozygous offspring from crosses of homozygous females and heterozygous males. (**B**) Loss of Hat1 in *Hat1*^*Δ57*^ mutants does not affect the daily motor activity, (**C**) total sleep time and (**D**) the number of sleep episodes per day. (**E**) *Hat1*^*Δ57*^ does not affect, while overexpression of *Hat1* increases female fecundity. The plots show the average number of eggs per female laid daily in vials containing *Hat1*^*rev16*^, *Hat1*^*Δ57*^, or *da-GAL4/P{EPgy2}Hat1*^*EY21697*^ (Hat1 OE) females mated with *w* males. (**F**) Loss of Hat1 does not affect heat-stress tolerance. The graph shows the percentage of 7-day-old *Hat1*^*rev16*^ (control) or *Hat1*^*Δ57*^ male or female flies that survived after a 25, 30 or 35 minutes heat-shock at 42 °C. (**G**) Heterozygous reduction of *Hat1* ameliorates mutant Huntingtin induced neurodegeneration. The graph shows the average number of rhabdomeres per ommatidium in the eyes of flies overexpressing a Httex1p-Q120 transgene and heterozygous for *Hat1*^*rev16*^ (control) or *Hat1*^*Δ57*^. On boxplots the bottom and the top of boxes indicate 25^th^ and 75^th^ percentiles, the line within the box shows the median value, whiskers represent 10^th^ and 90^th^ percentiles, dots mark outliers. Bar graphs show the averages, whiskers represent standard deviation. Asterisks mark significant differences compared to control, ***P < 0.001.

All recovered revertant lines, including *Hat1*^*Δ57*^, were homozygous viable and fertile proving that despite its fundamental role of H4 acetylation in early embryos *Hat1* is not an essential gene. To determine whether loss of Hat1 has subtler developmental or adult effects we set up several experiments. First, we made genetic crosses to determine whether loss of *Hat1* influences viability. We crossed *Hat1*^*Δ57*^ homozygous females to heterozygous males and by recording the number of homozygous and heterozygous F1 offspring per vials we found that the percentage of *Hat1*^*Δ57*^ homozygotes among the progeny was significantly lower (41.26 ± 5.92%, P = 4.77 * 10^−5^, Wilcoxon Signed Ranks Test) than that of heterozygotes (58.76 ± 5.92%) (Fig. 4A). Thus, we concluded that loss of *Hat1* results in a sub-lethal phenotype.

As Hat1 is not essential during development we aimed to characterize its potential effects on adult functions. It was reported previously that RNAi mediated Hat1 silencing resulted in circadian phase delay^19^. Therefore, we investigated whether loss of Hat1 affects the daily activity of flies since altered circadian activity may reflect disturbed circadian regulation or in more general terms dysfunction of the nervous and/or neuro-muscular system. We used a *Drosophila* Activity Monitor (TriKinetics Inc) to measure daily motor activity and sleep patterns of individual flies for five days under 12 hr light and 12 hr dark (12:12 LD) cycles. In our hands loss of Hat1 had no statistically significant effect on either the activity index (1.979 ± 0.424 vs 1.852 ± 0.426 counts/minute in *Hat1*^*rev16*^ vs *Hat1*^*Δ57*^, respectively, Fig. 4B), the total amount of daily sleep (794.4 ± 185.8 vs 817.9 ± 162.7 minutes/day in *Hat1*^*rev16*^ vs *Hat1*^*Δ57*^, respectively, Fig. 4C) or the number of daily sleep episodes (20.97 ± 6.27 vs 23.74 ± 7.06 in *Hat1*^*rev16*^ vs *Hat1*^*Δ57*^, respectively, Fig. 4D) of flies.

Next, as gene expression level of *Hat1* is highest in ovaries and early embryos^20^, we investigated whether *Hat1* affects female fecundity. We compared the average number of eggs laid daily by *Hat1*^*rev16*^, *Hat1*^*Δ57*^, and *da-GAL4/Hat1*^*EY21697*^ females, the latter of which overexpressing *Hat1*, and found significant differences (P = 3.66 × 10^−4^, Kruskal-Wallis Test) among these groups (Fig. 4E). Surprisingly, however, the lack of *Hat1* did not affect fecundity, the number of eggs laid per day by *Hat1*^*Δ57*^ homozygous females (21.83 ± 7.49) was similar to the amount seen in the *Hat1*^*rev16*^ controls (19.69 ± 7.81). On the other hand, females that over-expressed *Hat1* laid significantly more eggs per day (33.27 ± 11.37, P = 1.55 × 10^−4^, Wilcoxon Rank Sum Test) than control females. Thus, our data suggest that although *Hat1 per se* is not required for female fertility the biochemical functions it performs are rate limiting in egg production.

Next, we aimed to determine whether Hat1 plays a role in response to stress conditions in adults. First, we analyzed heat-stress tolerance of *Hat1* mutants. We heat-shocked 7-day-old *Hat1*^*rev16*^ and *Hat1*^*Δ57*^ male and female *Drosophila* at 40 °C for 25, 30 or 35 minutes and recorded the number of survivors after a 12-hour recovery period (Fig. 4F). Under these conditions the number of survivors significantly decreased with longer treatment times both in males and females (P < 0.0001 in both genders, Two-way ANOVA), however, we found no significant difference between the number of *Hat1*^*rev16*^ and *Hat1*^*Δ57*^ survivors in either gender.

We also tested another cellular stress condition, mutant Huntingtin induced proteopathy. Specific gain-of-function mutations of the human *huntingtin* (*HTT*) gene are responsible for Huntington’s disease (HD), a devastating neurodegenerative disorder^21^. In the mutant *HTT* allele a CAG triplet repeat is expanded resulting in an elongated polyglutamine region of the Huntingtin protein, which is primarily responsible for proteopathy in HD. We and others had previously shown that histone acetylation is perturbed in HD, however, the potential role of Hat1 in HD pathogenesis was not tested. We analyzed the effect of reduced *Hat1* in mutant Huntingtin induced neurodegeneration in the retina by the pseudopupil assay comparing the number of visible rhabdomeres, light gathering structures of photoreceptor neurons, per ommatidium. In wild-type flies each ommatidum contains 7 visible rhabdomeres. In the eyes of *elav-GAL4/w; HTTex1p-Q120/*+; *Hat1*^*rev16*^/+ flies expressing mutant *HTT* in the nervous system the average number of rhabdomeres per ommatidium was reduced to 5.83 ± 0.26, while in *elav-GAL4/w; HTTex1p-Q120/*+; *Hat1*^*Δ57*^/+ flies neurodegeneration was significantly (P = 4.35 × 10^−5^, Welch’s t-test) ameliorated with 6.39 ± 0.13 rhabdomeres per ommatidium (Fig. 4G).

### Transcription in the absence of Hat1

Several lines of evidence indicate that acetylation of nucleosomal histones positively correlates with gene expression^4^. Based on our data showing that Hat1 is responsible for the bulk of H4K5 and H4K12 acetylation in embryos we raised the question whether Hat1 is required for the proper regulation of gene expression. To investigate whether loss of Hat1 results in transcriptional dysregulation we compared gene expression levels of 6–12 hours old *Hat1*^*Δ57*^ mutant and *w*^1118^ (wild-type) control embryos by RNA-sequencing. In this developmental stage embryonic gene expression is already activated^22^.

**Figure 5:**
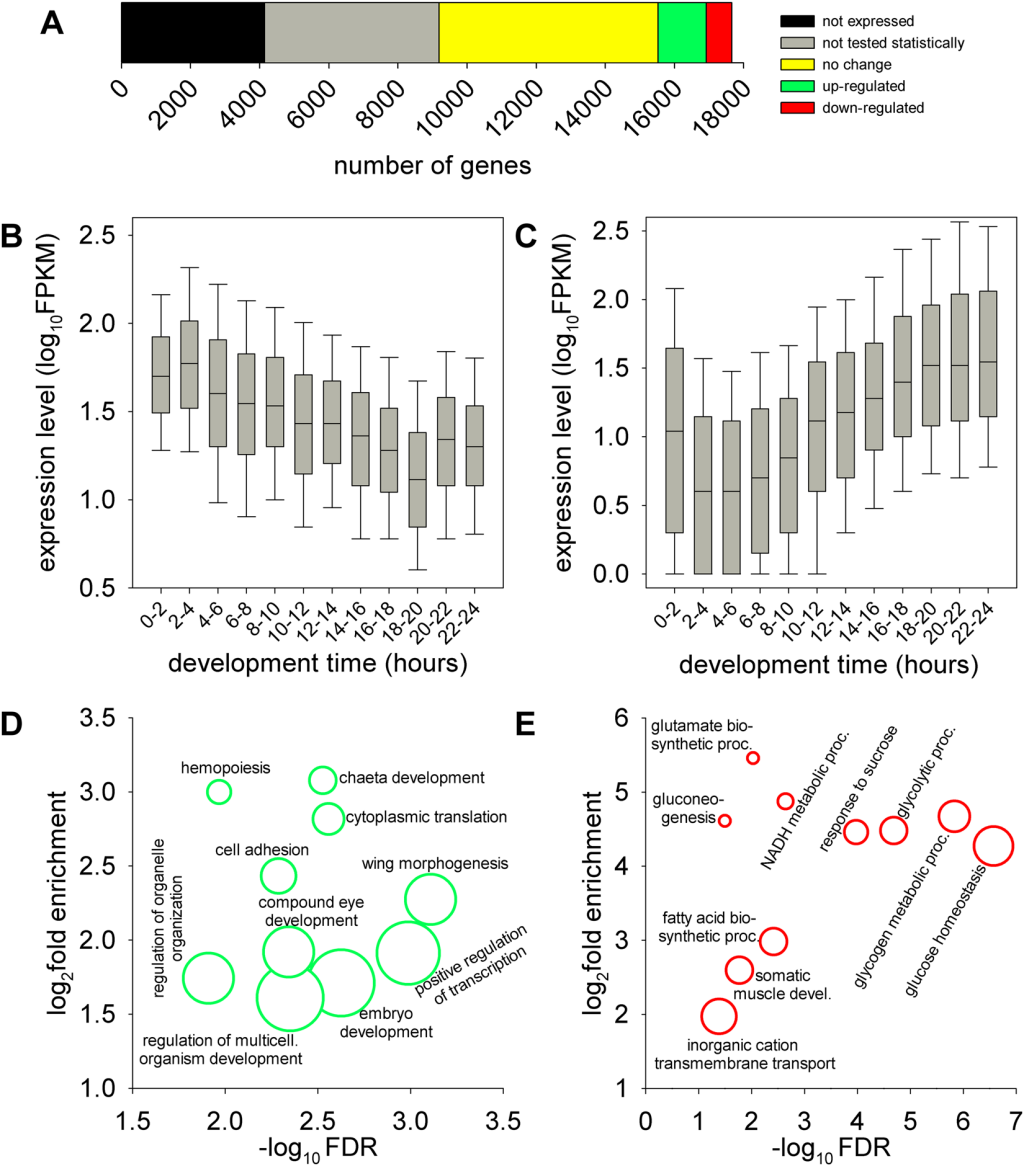
Transcriptomic changes in Hat1 mutants. (**A**) RNA-seq analysis of 6-12 hours old embryos showed that 7.9% of annotated genes were significantly (q < 0.05) up-regulated while 4.2% were down-regulated. The graph shows the number of genes that were not expressed (black), were expressed but not tested statistically (grey), did not show change in expression (yellow), were up-regulated (green) or down-regulated (red). (**B**) Transcriptional profile of genes up-regulated in 6-12 hours old *Hat1*^*Δ57*^ embryos during embryonic development based on modENCODE data. (**C**) Transcriptional profile of genes down-regulated in 6-12 hours old *Hat1*^*Δ5*^ embryos during embryonic development. Charts (**D,E**) show the ten specialized Gene Onthology Biological Process terms (from the bottom of the hierarchy) with the most significant false discovery rates (FDR) that showed enrichment among the 154 genes up-regulated and the 216 genes down-regulated in *Hat1*^*Δ57*^ embryos, respectively.

In the embryo derived samples 13518 of the 17661 annotated transcriptional units could be detected on the RNA level, 8471 of these could be statistically tested for differential gene expression. We found that the transcript level of 2137 (25.2% of statistically tested, 12.1% total) genes changed significantly (false discovery rate q < 0.05). Nearly two thirds (1397 genes, 65.4%) of these genes were up-regulated, while those making up the remaining one third (740 genes, 34.6%) were down-regulated (Fig. 5A). *Hat*1 itself was in the set of down-regulated genes with a Fragments Per Kilobase of transcript per Million mapped reads (FPKM) value of 1.5 in *Hat1*^*Δ57*^ samples compared to 46 in control samples, hereby validating the method and the samples. Thus, the majority of observed gene expression changes was in the opposite direction of what would have been expected as a result of reduced histone acetylation (i.e. reduced gene expression). This suggests that either a large portion of the observed effects are indirect or the acetylation by Hat1 is inhibitory to gene expression, possibly by augmenting chromatin formation.

To investigate whether loss of Hat1 can lead to the activation/derepression of inactive/silenced genes we compared the set of genes significantly up-regulated in *Hat1*^*Δ57*^ embryos to a set of genes not expressed in embryos (FPKM = 0) retrieved from *Drosophila* developmental modENCODE high-throughput RNA-seq dataset that contains embryonic transcriptional levels of annotated *Drosophila* genes with two hours resolution^20^. We found that out of the 3370 genes not expressed in embryos based on modENCODE data none was up-regulated in *Hat1*^*Δ57*^. Furthermore, in our RNA-seq dataset there were only 21 genes (0.98%) that suffered significant transcriptional changes in *Hat1*^*Δ57*^ and had a very low (FPKM < 3) expression level in the control samples. These data together suggest that loss of Hat1 alone is not sufficient for the activation or de-repression of inactive genes.

Next, we retrieved the embryonic transcriptional profile of genes showing significant expression changes in *Hat1*^*Δ57*^ embryos from the modENCODE developmental RNA-seq dataset^20^. We found that genes that were up-regulated or down-regulated in *Hat1*^*Δ57*^ embryos showed markedly different embryonic expression profiles (Fig. 5B,C). The expression levels of upregulated genes are higher in early embryos (with highest median expression level in 2–4 hours old embryos) and decrease as embryonic development progresses (Fig. 5B). As opposed to this, the expression levels of down-regulated genes are lower in early embryos (with lowest median expression level in 2–4 hours old embryos) and their expression levels increase during embryonic development (Fig. 5C). The characteristic transcriptional profiles of the two sets of dysregulated genes suggested that the majority of gene expression changes in *Hat1*^*Δ57*^ embryos could be explained by a delay in the transcriptional program that would cause the analyzed 6–12 hours old embryos having transcriptomic profiles similar to younger embryos. We selected those genes whose altered transcriptional levels in *Hat1*^*Δ57*^ embryos could not be explained by developmental delay by searching for up-regulated genes that have lower or equal expression levels between hours 0–6 than between hours 6–12 in wild-type embryos, and for down-regulated ones that have higher or equal expression levels between hours 0–6 than between hours 6–12. Altered transcript levels of 154 out of the 1397 up-regulated genes and 216 out of the 740 down-regulated genes could not be explained by developmental delay.

We used these subsets of dysregulated genes to identify the biological processes affected by loss of Hat1 by Gene Onthology (GO) term analysis. Overrepresentation test of biological process terms revealed that in the up-regulated gene set terms associated with development, translation and regulation of biosynthetic processes were enriched (Fig. 5D), while among the down-regulated genes biological process terms associated with cellular metabolism dominated (Fig. 5E). Thus, genes up- or down-regulated in *Hat1* mutant embryos are involved in distinct, well defined functions.

## Discussion

In this study we presented the phenotypic and molecular effects of a loss-of-function mutant of *Drosophila Hat1* for the first time. Although the deletion allele we generated does not remove the whole gene it removes ~80% of the coding sequence including the catalytic acetyltransferase domain rendering the mutant a functional null. By immunoblot analysis of *Hat1* mutants we confirmed that *Drosophila* Hat1, similarly to its orthologues, has histone H4K5 and H4K12 lysine specific acetyltransferase activities. However, Hat1 is not the only acetyltransferase in *Drosophila* showing this specificity. Previously it was reported that lack of the Ada2a subunit of the GCN5 acetyltransferase containing ATAC complex leads to reduced H4K5 and H4K12 acetylation in wandering L3 larvae^23^. Notwithstanding, our data shows that Hat1 is the dominant acetyltransferase with this substrate specificity in embryos as the level of acetylated H4K5 and H4K12 residues dropped to less than 4% of that of wild-type in *Hat1* mutant embryos. Importantly, this enzymatic activity seems to be directed to specific lysine residues as the acetylation state of another H4 N-terminal lysine, H4K8, was not affected by loss of Hat1. We also analyzed the acetylation levels of specific histone H3 residues as previously it was reported, that both steady state acetyl-H3K18 levels and H3K18 acetylation on histones deposited during replication was reduced in Hat1 mutant murine cells, while the level of H3K23 acetylation was not sensitive for the presence of Hat1^10^. Our results corroborate this data, we found that in *Hat1* mutant embryos H3K18 acetylation levels were significantly decreased compared to wild-type controls while there was no change in H3K23 acetylation. Thus, beside acetylation of histone H4 Hat1 also influences that of histone H3. However, as Hat1 has no H3 specific catalytic activity^15,17^ this is most likely an indirect effect.

The histone H4 K5/K12 diacetyl mark was proposed to be involved in the nuclear import of histones and chromatin assembly^24^, however, many questions remain about the potential role of this diacetyl mark and Hat1 in these processes. Histone H3 and H4 are transported to the nucleus with the aid of karyopherin proteins. More established models claim that H3:H4 form complexes with HAT1, the histone chaperone Asf1 and importin-4 (IPO4) in the cytoplasm^25^, while recently published data suggest that H3 and H4 might be imported to the nucleus by IPO4 as monomers where they associate with nuclear HAT1^26^. The nuclear localization signals (NLS) of histones H3 and H4 can be found at their positively charged N-terminal tails^27^. Analysis of the crystal structure of the *Kluyveromyces lactis* IPO4 homologue (Kap123) revealed that the H3-NLS and H4-NLS are competing for Kap123 binding: the H3K14, H3K23 and H4K16 residues are recognized by lysine binding pockets of Kap123^28^. Lysine to glutamine mutations of H4K16 or H4K5/K12 reduced the affinity of H4 towards Kap123, suggesting that positive charge at these positions facilitates karyopherin binding^28^. In *Saccharomyces*, mutation analysis of the H4 N-terminus in the absence of the H3-NLS showed that simultaneous mutations of H4 K5/K8/K12, and to a lesser extent, K5/K12 to glutamine resulted in cytoplasmic mislocalization of H4, while mutations to arginine did not have a similar effect^13^. However, deletion analysis of H3-NLS and H4-NLS indicated that in yeast the N-terminal tails of H3 and H4 are redundant for nuclear transport^13^. Furthermore, in *Hat1* null mouse embryonic fibroblast (MEF) cells there was no change in the level of either cytosolic or nuclear histone H4 suggesting that Hat1 dependent acetylation is not essential for the nuclear import of H4^29^. The data above suggest that while positive charge at the N-terminal tail of H4 promotes NLS function and its acetylation state influences nuclear transport of H4 specific post-translational modifications might play a redundant role in the import process. Our data corroborate these observations: we found that His4r proteins (which have the same amino acid sequence as H4) were localized to the nucleus in *Hat1*^*Δ57*^ mutants as well as when the His4r K5 and K12 residues were simultaneously mutated to arginine. In a recent article by Apta-Smith *et al*. the authors argue that Hat1 is mostly nuclear^26^ that would revert the timing of Hat1 dependent acetylation and nuclear import. Furthermore, as previous studies found that H4 K5/K12 acetylation rather impairs nuclear import than promotes it^13,28^ acetylation by Hat1 might be important for the release of H4 from the karyopherin inside the nucleus or binding to chromatin assembly factors that must precede the incorporation of H4 to chromatin.

Studies aimed at deciphering the role of H4 K5/K12 diacetylation in chromatin assembly also provided unambiguous results. In yeast, the entire N-terminal domains of both histone H3 and H4 in (H3-H4)_2_ tetramers were found to be dispensable for chromatin assembly factor-1 (CAF-1) dependent nucleosome formation on newly replicated DNA *in vitro*^12^ and mutation analysis also indicated that K5/K12 acetylation is not essential for nucleosome assembly^11^. Correspondingly, histone levels did not decrease on newly replicated DNA in *Hat1* null mouse cells^29^. However, H4K5 and K12 were found to be involved in Asf1 dependent chromatin re-assembly following DNA double-strand break repair in yeast^30^, while in chicken cells these residues were found to be required for correct centromeric localization of CENP-A as it was more frequently mis-incorporated to non-centromeric regions in H4-K5R-K12R point mutants^6^. Similarly, in *Drosophila* Hat1 is a subunit of one of the CENP-A loading complexes and its knock-down resulted in significantly reduced CENP-A incorporation into chromatin^17^. The above delineated data showing that H4 K5/K12 diacetylation contributes to but is not absolutely necessary for either nuclear transport or general chromatin assembly are in line with our results. We found that even though Hat1 is responsible for H4K5 and H4K12 acetylation in embryos it is a non-essential protein in *Drosophila*. Loss of *Hat1* did not affect fertility and only moderately reduced viability making it unlikely that Hat1 plays an essential, non-redundant role either in the transport of histones or in their assembly to chromatin. Redundancy in these processes can arise from several sources, including overlapping enzymatic activities, redundant acetylation positions on histones or redundant NLS functions of histone tails.

By analyzing the effects of loss of Hat1 on the transcriptome of 6–12 hours old embryos we found that more than two thousand genes were mis-regulated, nearly two-third of these genes were up-regulated while one-third were down-regulated. By cross-referencing our differential expression dataset with developmental RNA-Seq data of modENCODE we found that most of the genes up-regulated in *Hat1* mutant embryos have the highest expression levels in early embryos, while most of the down-regulated genes have the highest expression levels in older embryos. From this we assume that the majority of the transcriptional effects we detected in the absence of Hat1 are rather due to developmental delay caused by negative effects on replication and/or chromatin maturation than direct effects on the transcriptional machinery or its regulation. Hat1 dependent acetylation was implied to have a modulatory role in replication in human MCF7 cells as ATAD2 (ATPase family AAA domain-containing protein 2), a chromatin regulator shown to be required for proper chromatin localization of PCNA and efficient replication, binds H4 diacetylated at K5/K12 and is recruited to nascent chromatin^31^. Furthermore, nascent chromatin of *Hat1* null MEF cells are depleted of several bromodomain proteins while are enriched in Topoisomerase 2α and 2β suggesting that Hat1 has a role in chromatin maturation^29^. Defects in either of these processes might lead to the observed delay in the developmentally determined transcriptional program.

An interesting finding of this study is that reduced Hat1 ameliorates retinal neurodegeneration in a *Drosophila* model of Huntington’s Disease. In HD and in at least eight other neurodegenerative Polyglutamine (polyQ) disorders the primarily cause of pathogenesis is an elongated polyQ repeat in the disease proteins^32^. PolyQ containing aberrant proteins disturb important cellular processes, including epigenetic control of the genome^32^. Several factors affecting histone protein acetylation were tested previously with the conclusion that the activity of specific histone acetyltransferases (HAT) promoting open chromatin and gene expression and the level of histone acetylation are reduced in HD^33–36^, and that inhibition of histone deacetylases (HDAC) that remove acetyl marks and promote closed chromatin suppresses polyQ pathology^37–39^. However, not all acetylation related changes are linked to transcription or genome function, for example, inhibition of HDAC6 increases acetylation of α-tubulin and compensates for intracellular transport deficit observed in HD^40^. The effect of reduced Hat1 levels in *Hat1*^*Δ57*^ heterozygotes in the HD model is the opposite that was observed in the case of nuclear HATs^35,36^ suggesting that Hat1 might influence HD pathology by alternative means. First, instead of regulating transcription by acetylating nucleosomal histones on gene regulatory regions Hat1 might affect chromatin structure by modulating histone transport and deposition, as detailed above. Second, similarly to some other HAT enzymes Hat1 also has factor acetyltransferase activity i.e. its targets are not exclusively histones. For example, Hat1 regulates NF-κB signaling by acetylating the transcriptional regulator protein PLZF that leads to the recruitment of a histone deacetylase containing repressor complex^41^. At present there is a very limited body of knowledge about non-histone targets of Hat1 and detailed studies would be required to further the understanding of the role of Hat1 in the cell and the reasons of its evolutionary conservation.

## Methods

### *Drosophila* stocks and crosses

Stocks were maintained and crosses were done on standard cornmeal-yeast-agar *Drosophila* medium at 25 °C. The *y*^*1*^ *w*^*67c23*^*; P{EPgy2}Hat1*^*EY21697*^ strain was obtained from the Bloomington Drosophila Stock Center. To generate *Hat1* revertants *y*^*1*^ *w*^*67c23*^*; P{EPgy2}Hat1*^*EY21697*^ males were crossed to *w; Dr Δ2-3/TM3, Sb Δ2-3* females, then w; *P{EPgy2}Hat1*^*EY21697*^*/TM3, Sb Δ2-3* jump-starter male offspring were mated with *w; TM3/TM6* females. From the progeny of these crosses white eyed *w; Hat1*^*rev*^*/TM6* male revertants were selected and backcrossed individually to *w; TM3/TM6* females to generate revertant strains. *Hat1* revertant strains were genotyped by PCR analysis of the heterozygous *w; Hat1*^*rev*^*/TM6* founding males after they procreated.

For viability analysis, w; *Hat1*^*Δ57*^ females were crossed to *w; Hat1*^*Δ57*^/ZH-attP-86FB males in 20 vials, and the number of homozygous and heterozygous progeny that eclosed in each vial were recorded. Eclosion rates were calculated as the number of progeny of a given genotype divided by the number of all progeny in the vial.

For fertility assays, 3 days old *w*; *Hat1*^*Δ57*^, *w*; *Hat1*^*rev*^ or *da-GAL4* / *Hat1*^*EY21697*^ females were mated with *w*^*1118*^ males in 20 vials, 5 females and 3 males per vial. Vials were passed daily and the number of eggs laid in a 24 hour period was recorded.

For heat-stress tolerance assays freshly eclosed *Hat1*^*rev16*^ and *Hat1*^*Δ57*^ females and males were kept at 25 °C for 7 days then sorted to empty vials (20–25 flies / vial) and heat-shocked at 40 °C for 25, 30 or 35 minutes. After het-shock, flies were transferred to 25 °C and the number of survivors was scored after a 12 hours recovery period. At least 120 flies per treatment were tested.

To measure neurodegeneration *w; Httex1p-Q120* females were crossed with *elav-GAL4; Hat1*^*rev16*^*/Sb* or *elav-GAL4; Hat1*^*Δ57*^*/Sb* males and the *elav-GAL4/w; Httex1p-Q120/*+; *Hat1*^*rev16*^/+ and *elav-GAL4/w; Httex1p-Q120/*+; *Hat1*^*Δ57*^/+ progeny selected for pseudopupil assay.

### Activity monitoring

7-day-old *Hat1*^*rev16*^ and *Hat1*^*Δ57*^ male flies that were raised under 12:12 LD cycles were sorted to 5 mm diameter pyrex glass tubes (one fly per tube) and the activity of individual flies measured for five consecutive days under 12:12 LD condition at 25 °C in a DAM2 *Drosophila* Activity Monitor (TriKinetincs Inc). The DAM2 monitor records movement when a fly blocks a beam of infrared light hitting a sensor. A total of 38 flies per genotype were measured in three separate experiments. Monitor data were collected with DAMSystem and filtered with DAMFileScan programs (TriKinetics Inc) then analyzed with pySolo.

### Genotyping by PCR

Individual revertant males were homogenized in 50 μl homogenization buffer (10 mM Tris-HCl pH 8.0, 1 mM EDTA, 25 mM NaCl, 0.2 μg/μl Proteinase K), then incubated for 60 minutes at 37 °C followed by 15 minutes at 85 °C. 5 μl homogenates were used as templates in PCR reactions using Hat1.F and Hat1.R primers (Supplementary Table S1) and DreamTaq DNA polymerase (Thermo Fisher Scientific). Those revertants that gave a shorter amplicon than the 2 kbp one expected from *wild-type* were selected as deletion lines.

### Cloning and mutagenesis of *His4r* and generation of transgenic flies

*His4r* was cloned using nested primers. First, genomic region containing the *His4r* gene was PCR amplified from *w*^1118^ genomic DNA template with His4r.gF and His4r.gR primers (Supplementary Table S1) using Q5 High-Fidelity DNA polymerase (New England Biolabs) and cloned to pJET1.2 vector using CloneJET PCR cloning kit (Thermo Fisher Scientific) following the manufacturer’s recommendations. The gene body of *His4r* from the start codon to the codon before the stop codon was PCR amplified from the pJET1.2-His4r template using His4r.E3C.F and His4r.E3C.R primers (Supplementary Table S1) with Q5 DNA polymerase and reinserted to pJET1.2 with CloneJET PCR cloning kit. After digestion with FastDigest KpnI and EcoRI (Thermo Fisher Scientific) the His4r fragment was isolated and ligated to pENTR3C Gateway entry vector cut with the same enzymes.

K12R and K5R mutations were introduced to the His4r sequentially utilizing mutagenic PCR with modified primers. First, the His4r-K12R mutation was generated by PCR amplification of pENTR3C-His4r using Q5 DNA polymerase with His4r.K12R.F and His4r.K12.R primers (Supplementary Table S1). PCR fragments were isolated using Agencourt AMPure XP beads (Beckman Coulter), phosphorylated with T4 polynucleotide kinase (Thermo Fisher Scientific), circularized with T4 DNA ligase (Thermo Fisher Scientific) and template contamination removed by DpnI (Thermo Fisher Scientific) digestion. Next, K5R mutation was introduced similarly to pENTR3C-His4r-K12R construct using His4r.K5R.F and His4r.K5.R PCR primers (Supplementary Table S1) resulting in pENTR3C-His4r-K5R-K12R.

The inserts from pENTR3C-His4r and pENTR3C-His4r-K5R-K12R entry clones were subcloned to pTWFattB, a modified pTWF Gateway destination vector that contains a φC31 attB region cloned in the NsiI restriction site, with Gateway LR Clonase Enzyme Mix (Thermo Fisher Scientific) following the manufacturer’s protocol. Transgenic flies were generated by injecting the pTWFattB-His4r and pTWFattB-His4r-K5R-K12R constructs to embryos carrying the attP-zh86Fb φC31 docking site.

### Pseudopupil assay

7-day-old female flies were decapitated and their heads immobilized with nail polish on a microscope slide. The heads were covered with immersion oil and the number of visible rhabdomeres per ommatidium in the compound eye were counted using a Nikon Eclipse 80i microscope with a 50× Nikon oil immersion lens. At least 20 ommatidia per eye and at least 10 eyes per genotype were scored.

### Immunoblotting

For sample preparation *Drosophila* embryos were homogenized with a plastic pestle in sonication buffer (50 mM Tris-HCl pH7.9, 2 mM EDTA, 50 mM NaCl, 0.5 mM DTT, 10 mM Na-butyrate and 1x Protease inhibitor cocktail set I (Calbiochem)), then sonicated on “high” energy setting for four cycles of 30 sec ON/30 sec OFF in a Bioruptor sonicator (Diagenode). Homogenized samples were boiled for 10 minutes in 2x Laemmli sample buffer containing 5% β-mercaptoethanol, then centrifuged for 10 minutes at 13000 RPM. Sample supernatants containing 30 μg protein were separated by 10% Tris-Tricine-SDS PAGE and electrotransferred to Amersham Protran Premium 0.45 μm nitrocellulose membrane (GE Healthcare Life Sciences). Membranes were blocked in 5% nonfat milk and incubated with the following primary and secondary antibodies in the indicated dilutions: anti-acetyl-H4K5 (ab61236, Abcam, 1:500), anti-acetyl-H4K8 (ab15823, Abcam, 1:1000), anti-acetyl-H4K12 (ab61238, Abcam, 1:1000), anti-acetyl-H3K18 (ab1191, Abcam, 1:500), anti-acetyl-H3K23 (ab47813, Abcam, 1:1000) anti-H3 (ab1791, Abcam, 1:4000), goat-anti-rabbit IgG-HRP (P0448, Dako, 1:4000). Immunoblots were developed with Immobilon Western Chemiluminescent HRP substrate (Millipore) and recorded with a C-DiGit chemiluminescent blot scanner (Li-Cor Biosciences). Band intensities were quantitated with Image Studio software (Li-Cor Biosciences).

### Immunohistochemistry

Salivary glands of wandering L3 larvae were dissected, fixed for 20 minutes in fixative solution (4% formaldehyde in PBS), washed for 3 × 10 minutes in PBS then permeabilized for 1 hour in PBS containing 1% Triton X-100. After blocking for 50 minutes with 5% BSA in PBST (0.1% Tween 20 in PBS) samples were incubated at 4 °C overnight with monoclonal anti-FLAG M2 antibody (F3165, Sigma, 1:750) in PBST containing 1% BSA. After 6 × 10 minutes washes in PBST samples were incubated with goat anti-mouse IgG Alexa Fluor 488 secondary antibody (R37120, Thermo Fisher Scientific, 1:1500) for 1.5 hours at RT. After 4 × 10 minutes washes in PBST samples were stained with 0.5 μg/ml DAPI (PanReac AppliChem), mounted in Fluoromount (Sigma) and visualized with an Olympus FV10i Confocal Laser Scanning Microscope.

### RNA-sequencing

Total RNA samples were isolated from ~15 mg 6–12 hours old synchronized *w*^1118^ and *w*^1118^*; Df(2L)Hat1*^*Δ57*^ embryos with Qiagen RNeasy Plus Mini kit, three biological replicates each. Sample purity was assessed by measurement of OD_260/280_ ratio with a NanoDrop ND-1000 spectrophotometer; RNA concentrations were determined using fluorometric Qubit RNA HS Assay Kit (Thermo Fisher Scientific); and RNA integrity was verified by capillary gel electrophoresis with a Bioanalyzer 2100 (Agilent) instrument using Agilent RNA 6000 nano kit. RNA-seq libraries were prepared from 1 µg total RNA samples using TruSeq RNA sample prep kit v2 (Illumina) following the low sample (LS) protocol provided by the manufacturer. This, in short, includes purification and fragmentation of poly-A mRNA, synthesis of double-stranded cDNA with SuperScript II reverse transcriptase (Invitrogen), ligation of indexed sequencing adapters and limited amplification by PCR. Sequencing libraries were validated and average fragment length determined by capillary gel electrophoresis with a Bioanalyzer 2100 instrument using Agilent High Sensitivity DNA kit. Library concentrations were calculated based on average fragment length and quantitative-PCR measurement using NEBNext Library Quant Kit for Illumina (New England Biolabs). After denaturing libraries were diluted to 15 pM and were sequenced with an Illumina MiSeq sequencer using MiSeq Reagent Kit V3–150 providing 2 × 75 bp paired-end sequence reads.

### Sequencing data analysis

Primary data analysis (base-calling, fastq generation, demultiplexing) was done by MiSeq Control Software v2.6/Real-Time Analysis software v1.18.54. Fastq files were quality trimmed with Trimmomatic v0.33 in paired-end mode, then paired fastq files were aligned to the dmr6.13 *Drosophila melanogaster* reference genome with TopHat2^42^. Alignment files were sorted and deduplicated with SAMtools, then differential expression analysis was performed with Cufflinks^42^ using corresponding transcript annotation (dmr6.13.gtf) from FlyBase. Gene Ontology enrichment analysis was performed using PANTHER Overrepresentation Test (Released 20181003, Test type: Fisher’s Exact with FDR correction) with GO Ontology database (Released 2018-09-06) using all *Drosophila melanogaster* genes as reference list.

### Statistical methods

For statistical calculations R 3.4.3 was used. Data are given as mean ± standard deviation. We used Shapiro-Wilk test for normality testing and F-test to compare variances. In case of not normally distributed data Wilcoxon Rank Sum Test was used for pairwise comparison of unpaired samples, Wilcoxon Signed Ranks Test was used for pairwise comparison of paired samples, and Kruskal-Wallis Test was used for multiple comparisons followed by Wilcoxon Rank Sum Test with Bonferroni correction. For pairwise comparison of normally distributed data Student’s t-test was used if sample groups had equal variances while Welch’s t-test was used if sample groups had unequal variances. For multiple comparisons we applied ANOVA with post-hoc Tukey HSD test.

## Supplementary information

Supplementary  information 1

Supplementary  information 2

## Data Availability

RNA-Seq data are deposited to the National Center for Biotechnology Information Sequence Read Archive (NCBI SRA) under accession PRJNA551569. Other datasets used and/or analyzed during the current study are available from the corresponding author on reasonable request.
