# Supplementary material for: Hat1 acetylates histone H4 and modulates the transcriptional program in Drosophila embryogenesis
Source: Sci Rep. 2019 Nov 29;9:17973. doi: 10.1038/s41598-019-54497-0 (PMC6884459; doi:10.1038/s41598-019-54497-0)
Supplement: Supplementary file 1 — Supplementary information 1 [file 41598_2019_54497_MOESM1_ESM.docx]

**SUPPLEMENTARY MATERIAL**

**Hat1 acetylates histone H4 and modulates the transcriptional program in *Drosophila* embryogenesis.**

Júlia Varga^1,2^, Szabina Korbai^1,2^, Alexandra Neller^1^, Nóra Zsindely^1^, László Bodai^1,*^

^1^: Department of Biochemistry and Molecular Biology, Faculty of Science and Informatics, University of Szeged, 6726 Szeged, Közép fasor 52. Hungary

^2^: Doctoral School in Biology, Faculty of Science and Informatics, University of Szeged, 6726 Szeged, Hungary

*: to whom correspondence should be addressed: e-mail: bodai@bio.u-szeged.hu


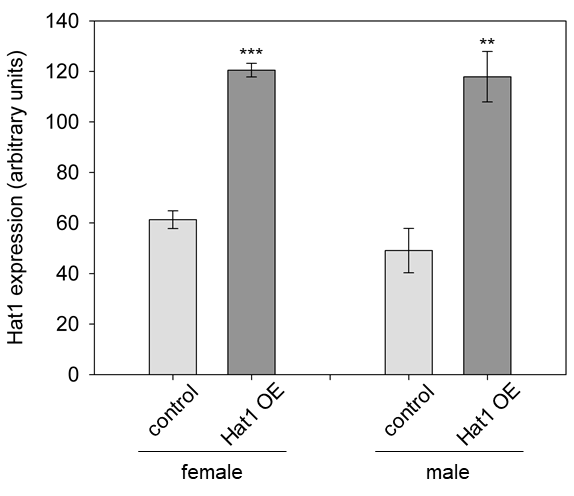


**Supplementary Figure S1. GAL4 dependent activation of the *P{EPgy2}Hat1^EY21697^* transposon insertion results in *Hat1* overexpression.**

For quantitative RT-PCR analysis total RNA was prepared from *w^1118^* (control) and *da-GAL4 / P{EPgy2}Hat1^EY21697^* (Hat1 OE) females and males with TRIzol (Invitrogen) reagent then RNA samples were treated with RNase-free DNaseI (Thermo Scientific) and first strand cDNA was prepared with TaqMan Reverse Transcription Reagents (Invitrogen) using random hexamer primers. qRT-PCR was performed in triplicates in a PikoReal Real-Time PCR System (Thermo Fisher Scientific) using Luna Universal qPCR Master Mix (New England Biolabs) with primers specific for the *Hat1* and *α-Tubulin at 84B* genes (Hat1.qF and Hat1.qR, and Tub.qF and Tub.qR, respectively, Supplementary Table S1). *Hat1* expression levels were calculated by setting threshold cycle (Ct) values against a template calibration curve and normalizing for the abundance of *α-Tubulin*. The graph shows averages of three biological replicates, error bars represent standard error of mean. *Hat1* transcript levels were significantly higher both in *da-GAL4 / P{EPgy2}Hat1^EY21697^* females (P = 0.00026, t-test) and males (P = 0.0069, t-test) than in control.


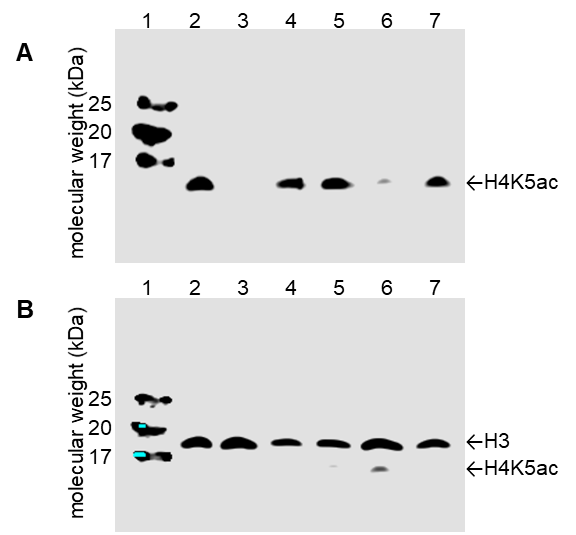


**Supplementary Figure S2.** **Immunoblot analysis of Hat1 mutants with anti-acetyl-H4K5 antibody.**

A. Lysates of *w^1118^* (lanes 2 and 5), *Hat1^Δ57^* (lanes 3 and 6) and *da-GAL4/+; Hat1^EY21697^/+* (lanes 4 and 7) embryos were run on a denaturing polyacrylamide gel and after electrotransfer the bottom part of the membrane was developed with anti-acetyl-H4K5 antibody (ab61236, Abcam, 1:500).

B. As loading control, the membrane shown on panel A was stripped under mild conditions to remove bound antibodies (stripping was not complete) and developed with anti-H3 antibody (ab1791, Abcam, 1:4000).

In lane 1 three bands of Triple Color Protein Standard III (SERVA) were labeled manually on the membrane with Glow-Writer Pen (LI-COR Biosciences). Immunoblots were developed with Immobilon Western Chemiluminescent HRP substrate (Millipore) and recorded with a C-DiGit chemiluminescent blot scanner (Li-Cor Biosciences).


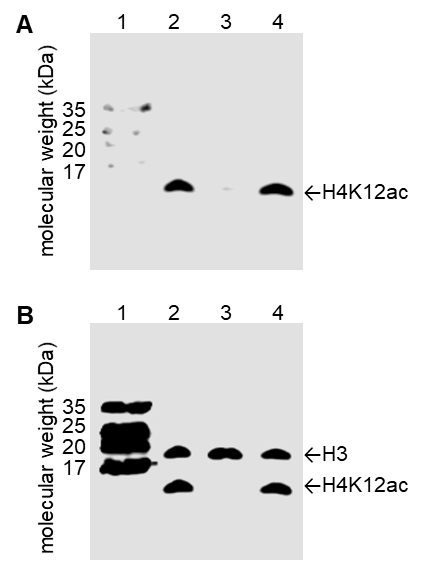


**Supplementary Figure S3.** **Immunoblot analysis of Hat1 mutants with anti-acetyl-H4K12 antibody.**

A. Lysates of *w^1118^* (lane 2) and *Hat1^Δ57^* (lane 3) and *da-GAL4/+; Hat1^EY21697^/+* (lane 4) embryos were run on a denaturing polyacrylamide gel and after electrotransfer the bottom part of the membrane was developed with anti-acetyl-H4K12 antibody (ab61238, Abcam, 1:1000).

B. As loading control, the membrane shown on panel A was stripped under mild conditions to remove bound antibodies (stripping was not complete) and developed with anti-H3 antibody (ab1791, Abcam, 1:4000).

In lane 1 four bands of Triple Color Protein Standard III (SERVA) were labeled manually on the membrane with Glow-Writer Pen (LI-COR Biosciences). Immunoblots were developed with Immobilon Western Chemiluminescent HRP substrate (Millipore) and recorded with a C-DiGit chemiluminescent blot scanner (Li-Cor Biosciences).


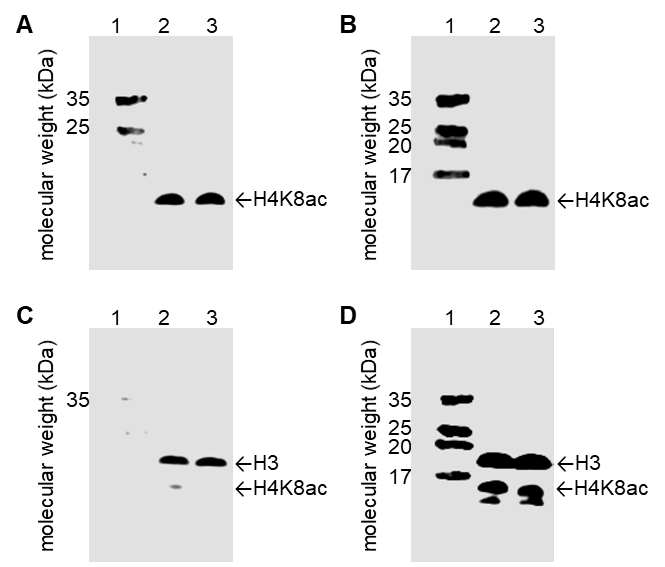


**Supplementary Figure S4.** **Immunoblot analysis of Hat1 mutants with anti-acetyl-H4K8 antibody.**

A. Lysates of *w^1118^* (lane 2) and *Hat1^Δ57^* (lane 3) embryos were run on a denaturing polyacrylamide gel and after electrotransfer the bottom part of the membrane was developed with anti-acetyl-H4K8 antibody (ab15823, Abcam, 1:1000).

B. Shows the same immunoblot as panel A with higher exposure settings.

C. As loading control, the membrane shown on panel A was stripped under mild conditions to remove bound antibodies (stripping was not complete) and developed with anti-H3 antibody (ab1791, Abcam, 1:4000).

D. Shows the same immunoblot as panel C with higher exposure settings.

In lane 1 four bands of Triple Color Protein Standard III (SERVA) were labeled manually on the membrane with Glow-Writer Pen (LI-COR Biosciences). Immunoblots were developed with Immobilon Western Chemiluminescent HRP substrate (Millipore) and recorded with a C-DiGit chemiluminescent blot scanner (Li-Cor Biosciences).


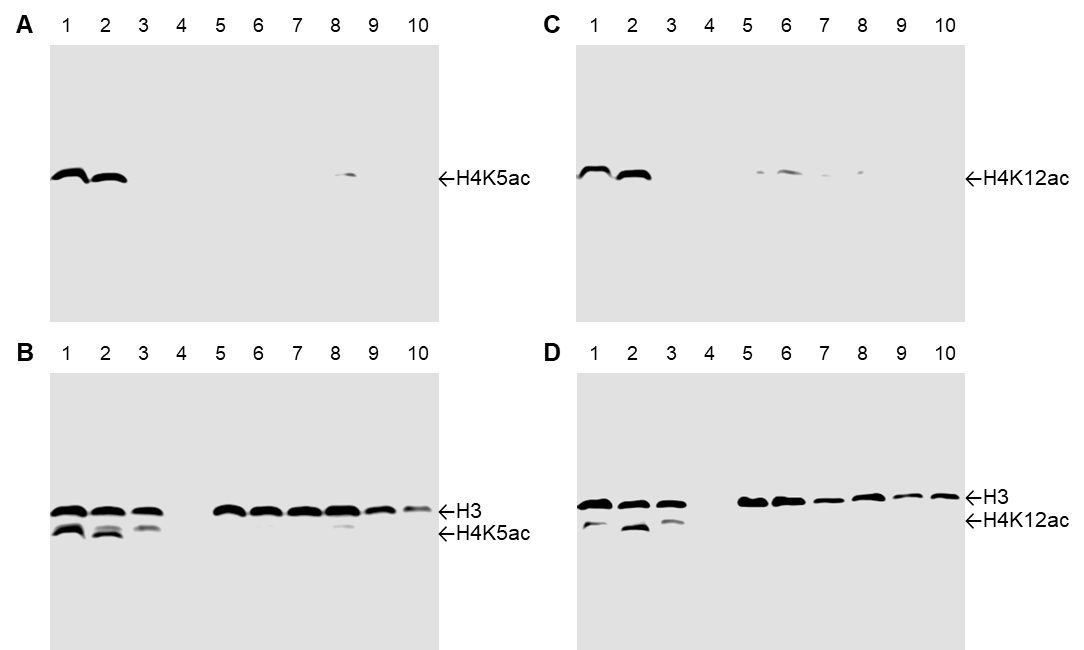


**Supplementary Figure S5.** **Immunoblot analysis of *Hat1* heterozygotes with anti-acetyl-H4K5 and anti-acetyl-H4K12 antibodies.**

As a control experiment we performed immunoblot analysis of *Hat1^rev16^* revertants and heterozygotes of *Hat1^Δ57^* and two other mutants generated by the imprecise excision of *P{EPgy2}Hat1^EY21697^*, *Hat1^Δ130^* and *Hat1^Δ184^*, that have ~1000 bp and ~600 bp deletions in the 5’ part of the *Hat1* gene, respectively.

A. Lysates of *Hat1^rev16^* (lane 1), *w* (wild-type, lane 2), homozygous *Hat1^Δ57^* (lane 3), *Hat1^Δ57^/Hat1^Δ130^* (lanes 5-8) and *Hat1^Δ57^/Hat1^Δ184^* (lanes 9-10) embryos were run on a denaturing polyacrylamide gel and after electrotransfer the bottom part of the membrane was developed with anti-acetyl-H4K5 antibody (ab61236, Abcam, 1:1000). Lane 4 was intentionally left empty.

B. As loading control, the membrane shown on panel A was stripped under mild conditions and developed with anti-H3 antibody (ab1791, Abcam, 1:4000).

C. Lysates shown on panel A were run on a denaturing polyacrylamide gel and after electrotransfer the bottom part of the membrane was developed with anti-acetyl-H4K12 antibody (ab61238, Abcam, 1:1000).

D. As loading control, the membrane shown on panel C was stripped under mild conditions and developed with anti-H3 antibody (ab1791, Abcam, 1:4000).

Immunoblots were developed with Immobilon Western Chemiluminescent HRP substrate (Millipore) and recorded with a C-DiGit chemiluminescent blot scanner (Li-Cor Biosciences).


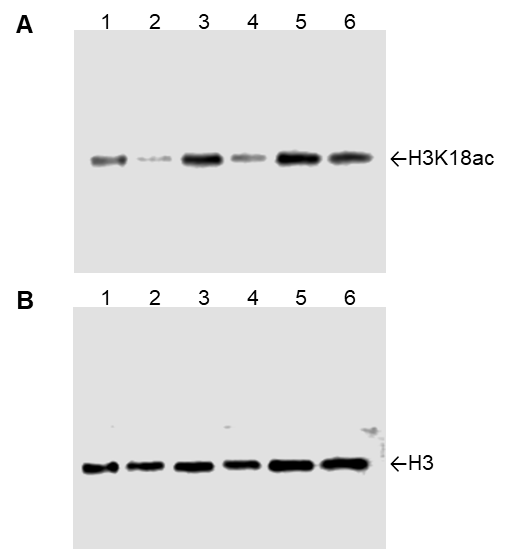


**Supplementary Figure S6.** **Immunoblot analysis of Hat1 mutants with anti-acetyl-H3K18 antibody.**

A. Lysates of *w^1118^* (lanes 1, 3 and 5) and *Hat1^Δ57^* (lanes 2, 4 and 6) embryos were run on a denaturing polyacrylamide gel and after electrotransfer the bottom part of the membrane was developed with anti-acetyl-H3K18 antibody (ab1191, Abcam, 1:500).

B. As loading control, the membrane shown on panel A was stripped under mild conditions to remove bound antibodies and developed with anti-H3 antibody (ab1791, Abcam, 1:4000).

Immunoblots were developed with Immobilon Western Chemiluminescent HRP substrate (Millipore) and recorded with a C-DiGit chemiluminescent blot scanner (Li-Cor Biosciences).


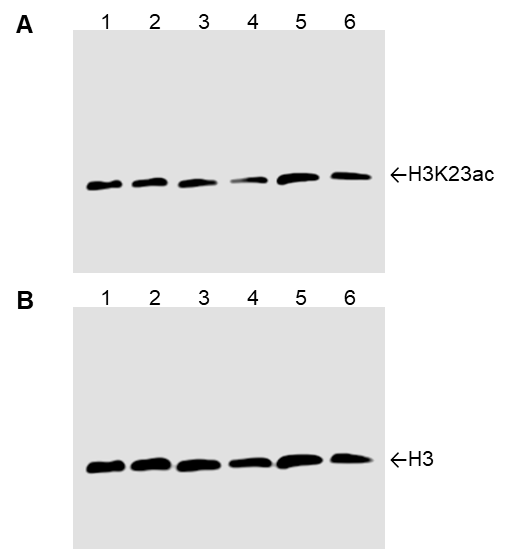


**Supplementary Figure S7.** **Immunoblot analysis of Hat1 mutants with anti-acetyl-H3K23 antibody.**

A. Lysates of *w^1118^* (lanes 1, 3 and 5) and *Hat1^Δ57^* (lanes 2, 4 and 6) embryos were run on a denaturing polyacrylamide gel and after electrotransfer the bottom part of the membrane was developed with anti-acetyl-H3K23 antibody (ab47813, Abcam, 1:1000).

B. As loading control, the membrane shown on panel A was stripped under mild conditions to remove bound antibodies and developed with anti-H3 antibody (ab1791, Abcam, 1:4000).

Immunoblots were developed with Immobilon Western Chemiluminescent HRP substrate (Millipore) and recorded with a C-DiGit chemiluminescent blot scanner (Li-Cor Biosciences).
