# Supplementary material for: Hat1 acetylates histone H4 and modulates the transcriptional program in Drosophila embryogenesis
Source: Sci Rep. 2019 Nov 29;9:17973. doi: 10.1038/s41598-019-54497-0 (PMC6884459; doi:10.1038/s41598-019-54497-0)
Supplement: Supplementary file 2 — Supplementary information 2 [file 41598_2019_54497_MOESM2_ESM.docx]

**SUPPLEMENTARY MATERIAL**

**Hat1 acetylates histone H4 and modulates the transcriptional program in *Drosophila* embryogenesis.**

Júlia Varga^1,2^, Szabina Korbai^1,2^, Alexandra Neller^1^, Nóra Zsindely^1^, László Bodai^1,*^

^1^: Department of Biochemistry and Molecular Biology, Faculty of Science and Informatics, University of Szeged, 6726 Szeged, Közép fasor 52. Hungary

^2^: Doctoral School in Biology, Faculty of Science and Informatics, University of Szeged, 6726 Szeged, Hungary

*: to whom correspondence should be addressed: e-mail: bodai@bio.u-szeged.hu

| **Name** | **Sequence** |
| --- | --- |
| Hat1.F | ACCAGAGCGAGATGTGTCAC |
| Hat1.R | AGAACCTACGAGGCCCTCAA |
| Hat1.qF | GTGGTGTTACTTCTTGAGCTACG |
| Hat1.qR | GGTAGCCAAGCCGAGTTTCT |
| His4r.gF | GGTACCGCACACACACATGGATTAAG |
| His4r.gR | GGTACCGGGAGAGCTAAATTTGCAG |
| His4r.E3C.F | GGTACCAAAATGACTGGTCGTGGAA |
| His4r.E3C.R | GAATTCGCACCGCCAAATCCGTA |
| His4r.K12R.F | AGGATTGGGACGCGGGGGCGCCAAG |
| His4r.K12.R | TTGCCTCCCTTTCCACGA |
| His4r.K5R.F | TGGTCGTGGACGCGGAGGCAAAGG |
| His4r.K5.R | GTCATTTTGGTACCGGATC |
| Tub.qF | TGTCGCGTGTGAAACACTTC |
| Tub.qR | AGCAGGCGTTTCCAATCTG |

**Table S1.: PCR primers used in this study.**
